# Health and equity: Venezuela’s and Brazil’s public health policies aimed at the LGBTQ+ population

**DOI:** 10.1093/eurpub/ckac131.511

**Published:** 2022-10-25

**Authors:** C Knihs de Camargo, S Moncada, N Nawal Huda

**Affiliations:** Global Public Health, Karolinska Institutet, Solna, Sweden

## Abstract

**Background:**

Public health policies aimed at the LGBTQ+ population within Latin America have been a theme of discussion among policymakers, governments, and society. Latin America’s conservative idiosyncrasy has delayed advances in this regard. A common factor has been inequity and difficulty in access to healthcare institutions by LGBTQ+ groups. Despite some improvements in some regions, there are still huge gaps between neighboring countries as in the case of Brazil and Venezuela. The aim of this study is to investigate and compare the existence and implementation of health policies aimed to improve the health equity of the LGBTQ+ population in both countries.

**Methods:**

Scoping review in international journal databases and grey literature, such as official government websites, and NGOs reports.

**Results:**

In Brazil, the “National Policy for comprehensive health of lesbians, gays, bisexuals and transgender populations” was created in 2011. Nonetheless, its implementation encounters challenges. The main difficulties are: under budgeting, lack of training of healthcare personnel, and challenges in monitoring and assessing proposed health interventions. In Venezuela, till today there is a lack of institutional protective policies and health policies directed at this population. Some examples include the impossibility to rectify documents to reflect transgender people’s identity, adequate protection from the law against civilian violations, same-sex marriage, and legal conditions for children of same-sex parents.

**Conclusions:**

The points presented above restrain satisfactory access to health care, positioning the LGBTQ+ population in a vulnerable setting, and making it a challenge to achieve health equity. In order to strengthen the health care systems in both countries, breakthrough measures need to be taken to assure fundamental human rights.

**Key messages:**

• Venezuela lacks basic institutional protective policies aimed towards LGBTQ+ population, while Brazil has a National LGBT Health Policy.

• Achieving health equity and assurance of rights to the LGBTQ+ population remains a challenge in both Brazil and Venezuela.

